# Involvement of distinct PKC gene products in T cell functions

**DOI:** 10.3389/fimmu.2012.00220

**Published:** 2012-08-06

**Authors:** Christa Pfeifhofer-Obermair, Nikolaus Thuille, Gottfried Baier

**Affiliations:** Division of Cell Genetics, Department of Pharmacology and Genetics, Medical University Innsbruck, Innsbruck,Tyrol, Austria

**Keywords:** T cell regulation, protein kinases, PKC isotypes, immune disease therapy

## Abstract

It is well established that members of the protein kinase C (PKC) family seem to have important roles in T cells. Focusing on the physiological and non-redundant PKC functions established in primary mouse T cells via germline gene-targeting approaches, our current knowledge defines two particularly critical PKC gene products, PKCθ and PKCα, as the “flavor of PKC” in T cells that appear to have a positive role in signaling pathways that are necessary for full antigen receptor-mediated T cell activation *ex vivo* and T cell-mediated immunity *in vivo*. Consistently, in spite of the current dogma that PKCθ inhibition might be sufficient to achieve complete immunosuppressive effects, more recent results have indicated that the pharmacological inhibition of PKCθ, and additionally, at least PKCα, appears to be needed to provide a successful approach for the prevention of allograft rejection and treatment of autoimmune diseases.

## INTRODUCTION

**FIGURE 1:**
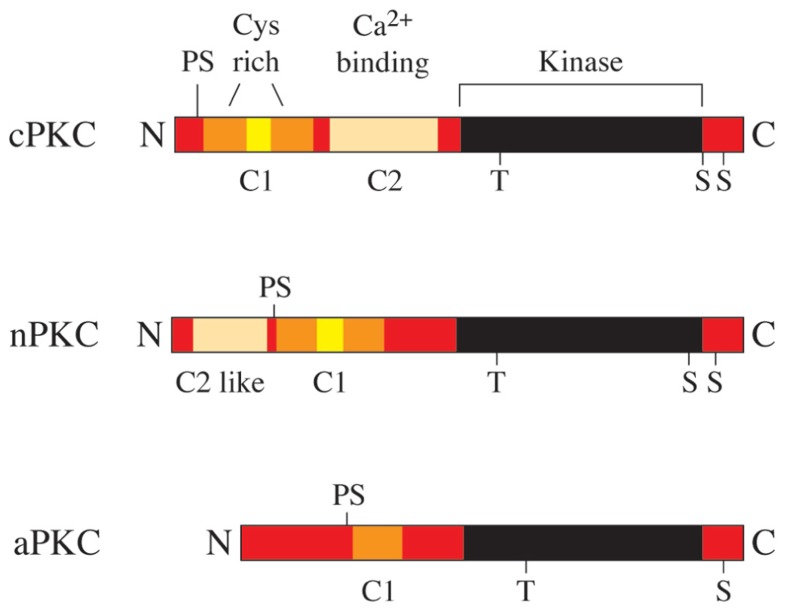
**The human PKC gene family.** PKC proteins are classified into conventional PKCs (cPKC; α, β, and γ), novel PKCs (nPKC; ε, δ, θ, and η) and atypical PKCs (aPKC; ζ and ι). cPKCs require Ca^2+^ and diacylglycerol (DAG) for activation, nPKCs are Ca^2+^ independent and aPKCs require neither Ca^2+^ nor DAG for activation.

**FIGURE 2:**
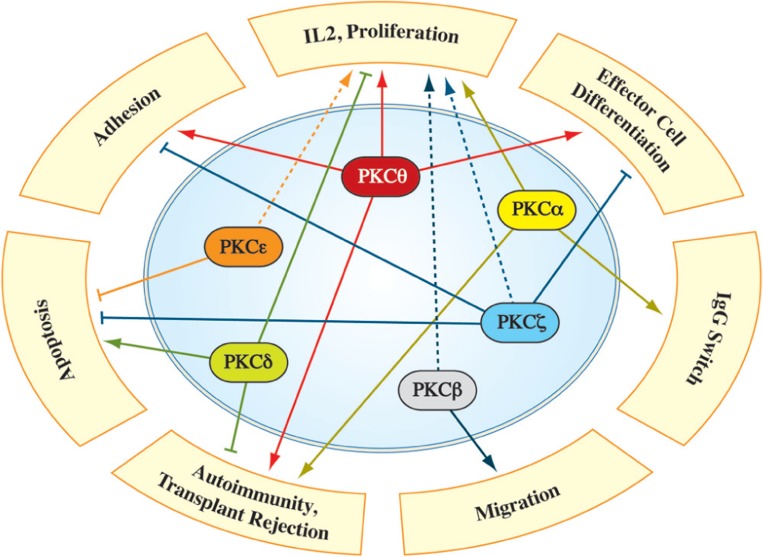
**Involvement of individual PKC family members in different aspects of T cell biology.** Numerous studies identified PKC isotype-selective functions in signaling pathways, necessary for full T cell activation, differentiation and robust immune responses *in vivo* (for details see text). The dashed line depicts PKC functions which were characterized primarily via overexpression/knockdown studies in immortalized cell lines, while a validation in a more physiological system is pending.

Members of the protein kinase C (PKC) family belong to the serine/threonine protein kinase subfamily, which plays an important role in the regulation of a variety of cell functions (**Figure 2**). The PKC family was originally discovered by Nishizuka and colleagues in 1977 (Takai et al., 1977) and consists of nine isotypes that are expressed in a wide range of cell types and tissues (**Figure 1**). The reasons for the heterogeneity of PKC isotypes are not yet fully understood. T lymphocytes, for example, express up to eight different species of PKC isotypes (**Table 1**), which makes it difficult to determine the specific cellular functions of these individual enzymes. The expression of more than a single PKC isotype in a given cell could suggest functional redundancy and/or specialization. **Table 1** summarizes the overall lymphoid expression patterns and T cell phenotypes of knockout T cells and the different PKC isotypes encoded in the human genome.

**Table 1:** Lymphoid expression pattern and immune cell phenotypes of PKC isotype knockout mice.

Gene loci	Tissue expression	Knockout mouse immune phenotype	Reference
**Conventional PKCs**
α	Ubiquitous, high in T cells	Reduced proliferation, reduced IFNγ production, defective IgG switching	Pfeifhofer et al. (2006)
β	Ubiquitous, high in B cells	Neutrophil-, B-, mast cell defect	Leitges et al. (1996), Nechushtan et al. (2000)
γ	Brain	ND
**Novel PKCs**
δ	Ubiquitous, high in T cells	Enhanced IL-2 secretion, enhanced proliferation, proapoptotic	Gruber etal. (2005), Lutz-Nicoladoni et al. (2005)
ε	Ubiquitous, high in T cells	Macrophage defect, defective bacterial clearance, influence on the nervous system	Castrillo et al. (2001), Kumar et al. (2002)
η	Ubiquitous, high in T cells	Impairment of epithelial regeneration in wound healing, increased susceptibility to tumor formation in skin carcinogenesis, defective homeostatic proliferation	Chida et al. (2003), Fu et al. (2011)
θ	T cells, platelets, monocytes	Reduced proliferation, reduced IL-2 production, abrogated AP-1, NF-κB, and NFAT transactivation, impaired EAE development, impaired T_H_2 immunity against *N. brasiliensis*	Sun et al. (2000), Pfeifhofer et al. (2003), Marsland et al. (2004), Salek-Ardakani et al. (2004), 2005
**Atypical PKCs**
ζ	Ubiquitous	Impaired T_H_2 cytokine secretion response	Martin et al. (2005)
ι	Ubiquitous	Lethal phenotype

## ROLE OF PKCθ IN IMMUNE CELL BIOLOGY

The main function of mature T cells is to recognize and respond to foreign antigens. This process involves the activation, adhesion, and differentiation of the resting cell into an effector lymphoblast that actively secretes immunoregulatory lymphokines or displays targeted cytotoxicity, ultimately leading to the recruitment of other cell types and initiation of an effective immune response. T cell activation is triggered by the ability of the T cell receptor (TCR) to recognize a peptide antigen, which is bound to major histocompatibility complex class I (MHCI) or class II (MHCII). T cells then begin to divide and differentiate on the basis of the processed antigen. The effector cell CD4^+^ T helper cell subset (including T_H_1, T_H_2, and T_H_17 cells) performs effector functions that are necessary to clear the pathogen. T_H_1 CD4^+^ T cells produce IFN-γ and IL-2 and promote cell-mediated immunity. T_H_2 CD4^+^ T cells produce IL-4, IL-5, IL-6, IL-10, and IL-13 and lead to the activation of the humoral immune system. T_H_17 CD4^+^ T cells produce IL-17, IL-21, and IL-22 and play roles in the defense against extracellular bacteria and fungi.

### INVOLVEMENT OF PKCθ IN THE IMMUNOLOGICAL SYNAPSE

After cell–cell contact between a T cell and an APC, this contact is stabilized during the initiation of an immune response by interaction of the β2-integrin LFA-1 with its counterligand ICAM-1 (Mazerolles et al., 1988; Dustin and Springer, 1989; Penninger and Crabtree, 1999). LFA-1 avidity is controlled by inside-out signaling via the control of integrin conformation and surface distribution (Lub et al., 1995; Carman and Springer, 2003; Dustin et al., 2004). One important inside-out signaling molecule that controls cell adhesion is the small GTPase Rap1 (Katagiri et al., 2002; Shimonaka et al., 2003). Rap1A-deficient T cells show impaired LFA-1 clustering and adhesion after CD3 stimulation (Duchniewicz et al., 2006). Letschka et al. (2008) found a role of a PKCθ/RapGEF2 complex in regulating LFA-1 avidity in T cells. These authors showed that after T cell activation, PKCθ phosphorylates RapGEF2 at Ser960, which regulates Rap1 activation and LFA-1 adhesiveness to ICAM-1. In agreement, this study showed that in OT-II TCR-transgenic CD4^+^ T cells, LFA-1 clustering after antigen activation was impaired in PKCθ-deficient CD4^+^ T cells (Letschka et al., 2008). According to their study, PKCθ seems to positively regulate the adhesive capacity of T lymphocytes.

When a stable contact between a T cell and an APC is formed, the T cell co-stimulatory receptor CD28 is activated by binding to its cell ligands CD80 or CD86. Subsequently, the immunological synapse is generated at the contact area between the T cell and the APC (Rao et al., 1999). Part of the immunological synapse is the supramolecular activation complex (SMAC), which is characterized by different signaling proteins, such as LCK (SRC family tyrosine kinase), LFA-1 (lymphocyte function-associated antigen 1), and CD45 (Freiberg et al., 2002). Effective T cell stimulation is characterized by the recruitment of PKCθ to the SMAC (Schaefer et al., 2004), at which it is phosphorylated by LCK at Tyr-90 (Liu et al., 2000). A physical interaction of PKCθ with the cytoplasmatic tail of CD28 has been shown to be essential in this recruitment mechanism (Kong et al., 2011). Subsequently, PKCθ is phosphorylated at different sites (Bauer et al., 2001; Bi et al., 2001; Liu et al., 2002; Freeley et al., 2005; Lee et al., 2005) and autophosphorylated at Thr-219 (Thuille et al., 2005). Recently, Chuang et al. (2011) identified the MAP4K3 GCK-like kinase (GLK) as a kinase that directly phosphorylates PKCθ at Thr-538 which is essential to activation of NF-κB in T cells. Phosphorylation is important to retain PKCθ in the immunological synapse, in which one of its functions seems to be the regulation of the immunological synapse itself. Through the live imaging of components of the immunological synapse, the synapse has been shown to be dynamic in wild-type mice but more stable in PKCθ-knockout mice, which influences the strength, duration and location of signals (Dustin, 2008).

### RECRUITMENT AND ACTIVATION OF SIGNALING MOLECULES

Another important role of PKCθ is to recruit and activate signaling molecules, such as phospholipase C (PLC), IL2-inducible T cell kinase (ITK), TEC, phospholipase C γ 1 (PLCγ1), and SPAK (a MAPKKK that ultimately activates AP1) to the immunological synapse. PKCθ was identified to play a critical role in the NF-κB and Ca^2+^/NFAT pathways to activate the IL-2 promoter. Antigen binding to the TCR leads to an increase in intracellular Ca^2+^, which activates calcineurin. Calcineurin dephosphorylates NFAT and leads to its nuclear import. Subsequently, NFAT forms complexes with the AP-1 protein transcription factor family and regulates the expression of IL-2 by binding to its promoter. PKCθ-knockout T cells were first described by Sun et al. (2000). They generated PKCθ-knockout mice by replacing the exon encoding the ATP-binding site of the kinase domain with the neomycin resistance gene. In their study they found strongly reduced proliferation of PKCθ^-/-^ CD3^+^ T lymphocytes accompanied by a reduced secretion of IL-2. Suitably they could show that TCR-initiated NF-κB activation was absent from PKCθ^-/-^ CD3^+^ T lymphocytes but was normal in thymocytes indicating that PKCθ is essential for TCR-mediated T cell activation (Sun et al., 2000).

Pfeifhofer et al. (2003) generated a conditional PKCθ-knockout mouse by using Cre-mediated recombination where the complete coding sequences of exons 3 and 4 are deleted, followed by a frame shift mutation between exons 2 and 5. Additionally to the results Sun et al. (2000) observed, they saw that a deficiency of PKCθ abrogates NFAT transactivation after CD3/CD28 stimulation. In addition, decreased intracellular Ca^2+^ flux was observed (Pfeifhofer et al., 2003).

To induce and maintain the complete IL-2-producing capacity of a T cell after TCR stimulation and activation of CD28, the RING (really interesting new gene)-type E3 ubiquitin ligase Cbl-b must be inhibited. Cbl-b restricts activation of the TCR by inhibiting the activation of PI3K (phosphoinositide-3-kinase; Fang and Liu, 2001) and PLCγ1 (Heissmeyer et al., 2004; Jeon et al., 2004), and it promotes the antigen-induced downregulation of the TCR (Naramura et al., 2002). In response to the stimulation of CD28, Cbl-b is ubiquitinylated and proteasomally degraded. Gruber et al. (2009a) showed that PKCθ directly regulates the ubiquitinylation and degradation of Cbl-b. After co-stimulation of the TCR and CD28, Cbl-b was degraded in wild-type CD3^+^ T cells but not PKCθ-deficient CD3^+^ T cells, and the ubiquitinylation of Cbl-b was strongly decreased after treatment with an inhibitor of PKCθ (Gruber et al., 2009a).

### *IN VIVO* IMMUNE RESPONSES

During T cell development, thymocytes undergo a twofold selection process. During positive selection, CD4^+^CD8^+^ double-positive thymocytes bearing TCRs with low or moderate affinity to MHC/antigen complexes expressed on epithelial cells receive a survival signal. During negative selection, the high-affinity interaction of TCRs with self-MHC/self-peptide complexes selects the thymocytes for apoptosis. Selected thymocytes downregulate CD4 or CD8 and leave the thymus as fully mature lymphocytes. To address the question of whether PKCθ is involved in positive selection, Morley et al. (2008) analyzed MHCII-restricted TCR-transgenic and non-transgenic PKCθ-knockout mice. In both mouse models, they found a severe defect in thymocyte positive selection (Morley et al., 2008). In agreement with these results, Gruber et al. (2010) also found a crucial role for PKCθ in the positive selection of thymocytes in a pathway leading to the activation of ERK, NFAT, and NF-κB by analyzing MHCI-restricted TCR-transgenic mice and non-transgenic PKCθ-knockout mice. When a naive CD4^+^ T cell is activated, it differentiates into the effector subsets T_H_1, T_H_2, or T_H_17. An imbalance of this differentiation leads to autoimmunity and hypersensitivity. Several studies showed that PKCθ is important in the regulation of the T_H_2-mediated immune response (Marsland et al., 2004; Salek-Ardakani et al., 2004, 2005; Tan et al., 2006). After infection with *Nippostrongylus brasiliensis*, T_H_2 cell immune responses were severely impaired in PKCθ^-/-^ mice. Consistent with these results, another *in vivo* study showed that PKCθ appears to be involved in lung inflammation responses, which are controlled by T_H_2 cells (Marsland et al., 2004; Salek-Ardakani et al., 2004). PKCθ^-/-^ mice develop drastically reduced pulmonary hypersensitivity responses to inhaled allergens, such as lung inflammation, eosinophil infiltration, and immunoglobulin E production.

To address the question of whether PKCθ is involved in protection against bacterial infections, Sakowicz-Burkiewicz et al. (2008) infected mice with *Listeria monocytogenes* (LM) and found that PKCθ is responsible for normal LM-specific T cell responses. Fauconnier et al. (2011) studied the role of PKCθ after the infection of mice with *Plasmodium falciparum*. They found that PKCθ-deficient mice are resistant to the development of cerebral malaria, and the recruitment and activation of CD8^+^ T cells in the brains of the resistant mice were reduced. To study the function of PKCθ in a chronic persisting infection model, Nishanth et al. (2010) infected mice with *Toxoplasma gondii*. PKCθ-deficient mice suffered from encephalitis and showed insufficient parasite control. *T. gondii*-specific CD4^+^ and CD8^+^ T cells were significantly reduced in the spleens and brains of infected PKCθ-deficient mice, indicating that PKCθ is important for intracerebral parasite control (Nishanth et al., 2010).

Tan et al. (2006) and Salek-Ardakani et al. (2004), 2005 showed that PKCθ is also important for full development of experimental autoimmune encephalomyelitis (EAE), a multiple sclerosis-like autoimmune disease that is T_H_17 dependent. PKCθ^-/-^ mice failed to develop EAE after injection with myelin oligodendrocyte glycoprotein (MOG). In addition, T_H_17 cells produced less IL-17 and failed to infiltrate the CNS.

Recently, Kwon et al. (2012) showed that PKCθ^-/-^ mice had lower levels of Stat3, a transcription factor required for T_H_17 differentiation, whereas the activation of Stat4 and Stat6, which are important for T_H_1 and T_H_2 differentiation was normal. Using a luciferase reporter gene driven by the Stat3 promoter they showed that PKCθ stimulates Stat3 transcription via the NF-κB and AP-1 pathway, resulting in the stimulation of T_H_17 differentiation (Kwon et al., 2012).

In striking contrast, PKCθ^-/-^ mice showed normal T_H_1 responses after infection with *Leishmania major* (Marsland et al., 2004), suggesting a lineage-specific function of PKCθ.

Garaude et al. (2008) found an impaired anti-leukemic response in PKCθ-deficient mice. These authors induced leukemia with Moloney-murine leukemia virus and found a higher disease incidence and a more rapid disease onset in PKCθ-knockout mice. Additionally, the intravenous injection of EL4 cells induced tumors earlier in PKCθ^-/-^ mice.

To avoid an uncontrolled immune response, the maintenance of the balance between immune tolerance to self-antigens and anti-tumor responses and the regulation of the suppression of effector T cells is mediated by regulatory T cells (T_reg_ cells; Sakaguchi et al., 2008). T_reg_ cells are produced in the thymus (nT_reg_) or from naive effector T cells (iT_reg_), and both types of T_reg_ cells express the transcription factor FoxP3, whereas nT_reg_ cells also express Helios (Zheng and Rudensky, 2007; Thornton et al., 2010). T_reg_ cells are able to suppress the function of CD4^+^ and CD8^+^ T cells, dendritic cells (DCs), NK cells, and B cells (Gupta et al., 2008b; Shevach, 2009). A deficiency of T_reg_ cells leads to multi-organ inflammatory diseases in mice (Sakaguchi et al., 2008). Gupta et al. (2008a) found a strongly reduced number of T_reg_ cells in PKCθ-knockout mice, but these cells were as potent as wild-type T_reg_ cells in inhibiting effector T cell activation, indicating that PKCθ was not required for T_reg_ cell-mediated inhibitory functions. However, Zanin-Zhorov et al. (2011) found that PKCθ was sequestered away from the T_reg_ immunological synapse with confocal imaging, and using a colitis mouse model and a poorly described PKCθ inhibitor, they postulated a PKCθ-mediated negative feedback loop that enhances the activity of human T_reg_ cells. A very recent publication by Ma et al. (2012) suggested that the differentiation of iT_reg_ cells is inhibited by PKCθ-mediated signals via the AKT-Foxo1/3A pathway.

## ROLE OF OTHER PKCs IN IMMUNE CELL BIOLOGY

### PKCδ

PKCδ is an isozyme belonging to a novel subclass of the serine/threonine PKC family and is expressed in most tissue and cell types. The kinase catalytic activity of PKCδ is mainly affected by trans- and autophosphorylation at conserved Ser/Thr sites in the catalytic domain (activation loop, turn motif, and hydrophobic motif), by tyrosine phosphorylation (by Src family kinases in the context of oxidative stress and DNA damage; Lu et al., 2007; Lomonaco et al., 2008) and by caspase-mediated proteolysis (during apoptosis; Kikkawa et al., 2002). Generally, upon stimulation, PKCδ translocates from the cytosol or nucleus to membrane/cytoskeletal compartments, enabling the phosphorylation of many target proteins and leading to the activation of several signal transduction pathways. It has also been shown that PKCδ can shuttle to mitochondria (Li et al., 1999; Majumder et al., 2001). PKCδ negatively affects a wide variety of cellular functions by inhibiting cellular growth and proliferation and promoting cell death, but it has also been shown to contribute to mitogenesis (Watanabe et al., 1992; Nakagawa et al., 2005; Santiago-Walker et al., 2005), migration (Jackson et al., 2005), differentiation (Cerda et al., 2001; Yang et al., 2006; Zhang et al., 2008), and tumor progression. Different studies have revealed a role for PKCδ in the initiation, progression, and maintenance of inflammatory processes by affecting NF-κB transactivation (Satoh et al., 2004; Hsieh et al., 2007).

Additionally, a pro-apoptotic role for PKCδ has been described in T cells. The subcellular localization of PKCδ in human T cells during apoptotic induction by cytokine deprivation and Fas ligation and during the prevention of apoptosis by IFNβ addition was analyzed by Scheel-Toellner et al. (1999). The addition of INFβ to T cells in a pro-apoptotic environment led to a rapid re-translocation of PKCδ from the nucleus and inhibited the caspase-3-mediated proteolytic activation of PKCδ (Scheel-Toellner et al., 1999). An essential role for PKCδ in the apoptotic induction of mouse thymocytes was addressed in a study by Lutz-Nicoladoni et al. (2005). Thymocytes from a large panel of PKC-knockout mice were forced to undergo apoptosis *in vitro* via treatment with different apoptotic inducers (PDBu, dexamethasone, FasL, staurosporine, or etoposide), and the selective involvement of PKC isotypes in this process was assessed. PKCδ-deficient primary mouse double-positive thymocytes were protected from apoptotic induction, indicating a clear pro-apoptotic role of PKCδ (Lutz-Nicoladoni et al., 2005). Gruber et al. (2005) investigated the proliferative response and IL-2 cytokine secretion of PKCδ-deficient CD3^+^ T cells versus control cells *in vitro* via allogenic MHC stimulation and *in vivo* via injection of anti-CD3 antibodies. The significantly enhanced proliferation and IL-2 cytokine production of mature T cells and the increased blood plasma IL-2 levels in PKCδ-null mice led to the assumption that PKCδ acts as a negative regulator of T cell activation responses (Gruber et al., 2005).

An involvement of PKCδ in lytic granule exocytosis of CD8-CTLs (cytotoxic T lymphocytes) was shown by Ma et al. (2007), 2008. The combined use of pharmacological inhibitors and mice with targeted gene deletions allowed these authors to demonstrate that PKCδ is selectively required for lytic granule movement in response to TCR engagement on CD8^+^ CTLs but is dispensable for activation, cytokine production, and the expression of cytolytic molecules in response to TCR stimulation. In a follow-up study, the authors showed via a time-lapse analysis of living CD8^+^ CTLs that PKCδ localizes to secretory lysosomes and accumulates at the immunological synapse during target killing (Ma et al., 2007, 2008).

A correlation between impaired PKCδ activation/ phosphorylation and the development of idiopathic and hydralazine-induced lupus was postulated by Gorelik et al. (2007). PMA-stimulated CD4^+^ T cells from patients with lupus showed an impaired PKCδ activity state compared with CD4^+^ T cells from healthy donors. This defect was responsible for decreased ERK signaling and led to increased CD70 expression due to insufficient demethylation of the CD70 promoter (Gorelik et al., 2007).

The expression level and activity state of PKCδ and PKCζ was investigated in amyloid β1–42 (Aβ1–42)-reactive T cell populations in Alzheimer disease (AD) patients in comparison to healthy individuals. This study clearly showed the increased expression and activation of PKCδ in Aβ-stimulated peripheral T cells from early AD patients, whereas the same treatment induced two distinct (p)PKCδ and (p)PKCζ T cell subpopulations in severe AD patients (Miscia et al., 2009).

### PKCε

PKCε was first discovered among the novel PKC isotypes and is expressed at high levels in neuronal, hormonal, and immune cells. Essential roles for PKCε have been established in numerous cellular functions, including proliferation, differentiation, gene expression, muscle contraction, transport, tumorigenesis, exocytosis, and endocytosis. In addition to the classical activation by auto- and trans-phosphorylation at conserved sites in the catalytic domain, PKCε is activated by several different second messengers, including diacylglycerol (DAG), phosphatidylinositol-3,4,5-triphosphate, and fatty acids. PKCε is targeted to specific cellular compartments depending on the interaction of second messengers with its C1 domain (DAG and tridecanoic acids evoke a plasma membrane and/or cytoskeleton translocation, whereas arachidonic and linoleic acids lead to recruitment to Golgi networks) and via crosstalk with adaptor proteins (i.e., Rack1 and β-Cop). An association of PKCε (via its actin-binding motif) with actin filaments in response to phosphatidylserine-independent stimulation has been reported (Akita, 2002).

In T cells, numerous studies have directly shown a positive role of PKCε in the regulation of NF-κB/NFAT/AP1 pathways leading to IL-2 upregulation; the activation-dependent translocation of PKCε from the cytosol to the membrane compartment in TCR/CD3- or PMA-stimulated human PBLs has been reported previously (Keenan et al., 1997). The neutralization of PKCε in this cell type via the introduction of antagonistic antibodies led to a downregulation of IL-2 synthesis (Szamel et al., 1998). Jurkat T cells expressing a constitutively active PKCε mutant showed increased AP1 and NFAT1 transactivation (Genot et al., 1995). An inhibitory effect of eicosapentaenoic acid (EPA) and docosahexaenoic acid (DHA) in the plasma membrane translocation of PKCε (and PKCα), NF-κB nuclear translocation, and IL-2 transcription in PMA-stimulated Jurkat T cells has been described (Denis et al., 2005). A pivotal role for PKCε in thrombin-mediated ERK1/2 activation in Jurkat cells has been shown by Maulon et al. (2001). The poor ability of neonatal T cells to produce lymphokines was linked to a lower PKCε (and PKCβ, PKCθ, and PKCζ) expression level in this cell type, which is correlated with an activation defect of MAPK pathways (Hii et al., 2003).

Interestingly, Gruber et al. (2005) reported that mice carrying a homozygous disruption of the PKCε locus showed unaltered T cell development and maturation; in addition, mature primary CD3^+^ T cells isolated from PKCε^-/-^ mice showed normal proliferation, IL-2 secretion responses, and NF-κB transactivation upon CD3/CD28 stimulation or allogeneic MHC presentation, suggesting that PKCε loss of function is compensated for by other members of the PKC family. In contrast to the described redundant function of PKCε in mouse T cell proliferation, a role of the PKCε isotype in the regulation of human CD4^+^ T cell proliferation and sensitivity to TGFβ1 has been shown by Mirandola et al. (2011). PKCε silencing by siRNA led to decreased IL-2 receptor chain expression and proliferation and reduced NF-κB1 and NF-κB2 gene expression upon CD3/CD28 stimulation, whereas the inhibitory effects of TGFβ1 were potentiated by PKCε downregulation. In addition, a possible connection between increased PKCε expression levels in CD4^+^ T cells from Hashimoto thyroiditis patients and the molecular pathophysiology of this autoimmune disease was postulated (Mirandola et al., 2011).

Some studies have identified an anti-apoptotic role for PKCε: Jurkat T cells were rescued from Fas-mediated apoptosis by PKCε via the p90Rsk-dependent phosphorylation and inactivation of BAD (Bertolotto et al., 2000). The basis for the deletion of autoreactive thymocytes during negative selection was previously addressed (Simon et al., 2000); a lack of the constitutive expression of PKCε in antigen-stimulated CD4^+^/CD8^+^ thymocytes (in comparison to mature T cells) leading to an inhibition of NF-κB activity and increased cell death was postulated as a probable cause.

A positive involvement of PKCε in the recovery of downregulated sphingosine-1-phosphate receptor 1 (S1PR1) in primary mouse CD4^+^ T cells was investigated (Graeler et al., 2003) in PKCε-null mice and with PKCε-selective inhibitors.

Quann et al. (2011) established a new redundant role for PKCε and PKCη in T cell polarity; the photoactivation of TCR induced a rapid accumulation of both PKC isotypes in a broader domain of the plasma membrane, in which they were required to promote the recruitment of PKCθ to the center of the immunological synapse and subsequent microtubule-organizing center (MTOC) reorientation.

### PKCζ

PKCζ is a calcium- and diacylglycerol-independent serine/threonine protein kinase that belongs to the atypical subfamily of PKC isoforms and displays strong homology (more than 70%) to PKCι/λ. It is ubiquitously expressed but is more highly expressed in the lung, brain, and testis. PKCζ contains a PB1 domain in the N-terminus that recognizes OPCA (OPR/PC/AID) motifs of other proteins, such as the scaffold proteins PAR-6 and ZIP/p62 and the kinase MEK5. PKCζ activity is regulated by PDK-1 transphosphorylation of the catalytic domain activation loop, autophosphorylation, and important lipid components, such as phosphatidylinositols, phosphatidic acid, arachidonic acid, PIP3, and ceramide. Prostate apoptosis response-4 (Par-4) and partitioning defective gene-3 (PAR-3) have been reported to inhibit PKCζ activity through a specific protein–protein interaction. PKCζ has been shown to be involved in the regulation of several critical pathways for cell survival, proliferation, differentiation, and cell polarity, thereby affecting the NF-κB and MAPK pathways. A special role in modulating translation via the p70S6 kinase signaling cascade has also been described by numerous studies (Hirai and Chida, 2003). Recently, a link between PKCζ activity and TGFβ receptor trafficking and degradation has been shown (Gunaratne et al., 2012).

The activation of the PKCζ isotype has been shown to be an important step in the IL-2-mediated proliferation of T cells and in maintaining the integrity of the actin cytoskeletal structure (Gomez et al., 1995). Furthermore, an association between PKCζ and PI3K has been reported to be necessary for the phosphorylation/activation of PI3K in IL-2-stimulated TS1-α/β mouse T cells (Gomez et al., 1996). Through the transient overexpression of wild-type or a dominant-negative mutant of PKCζ in Jurkat T cells, a previous study (San-Antonio et al., 2002) observed that PKCζ can phosphorylate NFAT and regulate its activation status. Additionally, an involvement of both PKCζ and PI3K in NF-κB/c-Rel transactivation regulation in TNFα-stimulated Jurkat T cells was postulated (Martin et al., 2001). A previous study (Sanchez-Valdepenas et al., 2007) addressed the effect of TCR/CD28 co-stimulation on the inducible phosphorylation/transactivation of the NF-κB members p65/RelA and c-Rel. Cot kinase, PKCζ, and NF-κB-inducible kinase (NIK) seemed to be involved in potentiating c-Rel transactivation activity through the phosphorylation of a restricted set of Ser residues, whereas NIK seemed to be unnecessary for the activation of p65. Additionally, Gruber et al. (2008) found a physical and functional interaction between PKCζ and the novel PKCθ isotype in the NF-κB activation of Jurkat T cells. A stimulation-dependent colocalization of the PKCζ/ι–PKCθ complex to lipid rafts was monitored via confocal microscopy. However, peripheral CD3^+^ T cells isolated from the spleen and lymph nodes of PKCζ-deficient mice showed normal proliferation and IL-2 cytokine responses to CD3/CD28 activation, indicating a possible functional redundancy with PKCι/λ, the closest structural relative (Gruber et al., 2008).

A critical role for PKCζ in IL-4 signaling and T_H_2 differentiation *in vitro* and *in vivo* has been reported (Martin et al., 2005). PKCζ-deficient CD4^+^ T cells showed an impaired secretion of T_H_2 cytokines and a defective Stat6/Jak1 pathway. Moreover, PKCζ^-/-^ mice were protected from ovalbumin-induced T_H_2-driven allergic airway disease in an asthma model.

A protective role for PKCζ against FasL-induced apoptosis was previously described (Leroy et al., 2005); PKCζ interfered with FADD recruitment to the death-inducing signaling complex (DISC) and subsequent caspase-8 processing.

PKCζ has been shown to act in combination with nitric oxide synthase (NOS) in the regulation of thyroid hormone (TH)-mediated T cell proliferation (Barreiro Arcos et al., 2006); TH treatment increased atypical PKCζ expression and NOS activity, whereas PKCζ inhibition abrogated the basal and TH-induced activation of NOS.

A role for PKCζ in the biological processes of adhesion and cell motility has been described by several studies. The mechanism of the CD4-triggered regulation of LFA-1-mediated adhesion was investigated (Trucy et al., 2006). CD4 binding increased the activity of both PDK1 and PKCζ, and both kinases were necessary for the downregulation of LFA-1-dependent adhesion in the A201-CD4^+^ T cell line in a PI3K-dependent manner. Real et al. (2007) showed that PKCζ and PKCι were both required for T cell motility and the ability to scan DCs downstream of chemokine receptors.

### PKCη

PKCη is classified into the novel PKC subfamily and shows a high sequence similarity to PKCε. It was originally isolated from a cDNA library of mouse skin in 1990 (Osada et al., 1990) and is localized on human chromosome 14 (Quan and Fisher, 1999) and mouse chromosome 12 (Chida et al., 1998). It is predominantly expressed in squamous epithelia including skin, tongue, esophagus, and trachea (Koizumi et al., 1993), but at high levels also in T and B cells (Mischak et al., 1991). In addition to phosphatidylserine and diacylglycerol, PKCη can be specifically activated by cholesterol sulfate (Ikuta et al., 1994). An involvement in keratinocyte cell growth, terminal differentiation, and cell cycle arrest has been reported by several studies: PKCη was shown to associate with and to activate Fyn, leading to keratinocyte growth arrest and differentiation (Cabodi et al., 2000); a PKCη induced terminal differentiation through a transcriptional activation of TGas1 and involucrin was described by Ueda et al. (1996) and Efimova and Eckert (2000). In addition, PKCη has been shown to induce G1 arrest in keratinocytes via an inhibition of cyclin-dependent kinase 2 activity (Kashiwagi et al., 2000). An important role in the regulation of cell division and cell death during early B cell development was postulated by the work from Morrow et al. (1999).

The different lipid raft localization pattern of PKCα, PKCη, and PKCθ in cisplatin-induced apoptotic Jurkat T cells was investigated by Solstad et al. (2010). A selective upregulation of PKCα in these microdomains upon apoptosis induction was revealed, whereas the levels of PKCη and PKCθ were significantly reduced.

Recently, Fu et al. (2011) found a pivotal role of PKCη in T cell activation and homeostatic proliferation. Comparing the phenotypes of PKCη^-/-^, PKCθ^-/-^, and mice with a targeted disruption of both PKC isoforms, they were able to show that both isoforms share some redundancy in T cell biology. Both isoforms are recruited to the immunological synapse upon TCR stimulation and double-knockout mice showed a more severe defect in positive selection. Additionally, they found specific non-redundant functions as in self-antigen-dependent homeostatic proliferation. Using a live imaging approach a TCR-induced recruitment of GFP fusion proteins of PKCη and PKCε to the plasma membrane was also described by Quann et al. (2011). The timely well coordinated localized enrichment of these two isoforms served as a prerequisite for the subsequent translocation of PKCθ to the center of the immunological synapse, necessary for the regulation of T cell polarity and T cell effector functions.

### PKCβ

The alternative splicing forms PKCβI and PKCβII are members of the calcium-activated, phospholipid- and DAG-dependent classical or conventional PKC subfamily. Numerous studies have shown their role in various cellular processes, such as the regulation of B cell development and activation/proliferation, oxidative stress-induced apoptosis, androgen receptor-dependent transcription regulation, insulin signaling, and endothelial cell proliferation. In B cells, a signaling link between PKCβ and BTK has been described; PKCβ can downregulate BTK function through the direct phosphorylation of BTK at Ser-180, inhibiting its membrane translocation and subsequent activation (Kang et al., 2001). A key role for PKCβ in BCR-induced NF-κB activation has been shown (Sommer et al., 2005); the direct phosphorylation of CARMA1 at three serines within its linker region induced its translocation into lipid rafts, the recruitment of BCL10/Malt1 and the subsequent activation of signaling molecules downstream of the CBM complex. Furthermore, PKCβ seems to play an important, even dual role in insulin signaling pathways: in muscle cells, PKCβ mediates insulin-dependent DNA synthesis through the RAF1-MAPK/ERK signaling cascade downstream of insulin receptor substrate 1 (IRS1), and in adipocytes, it negatively regulates glucose transport by inhibiting the translocation of the glucose transporters GLUT1 and GLUT4 (Formisano et al., 2000; Bosch et al., 2003; Perrini et al., 2004).

A selective impact of PKCβ on T cell migration has been shown by several studies (Volkov et al., 1998, 2001). LFA-1-triggered T cell locomotion led to the specific recruitment of PKCβ and PKCδ to the MTOC and microtubules. A PKCβ-deficient T cell line was unable to either crawl or develop a polarized microtubule array upon integrin cross-linking, whereas the ability to adhere and form actin-based pseudopodia remained unaffected. The reconstitution of PKCβ(I) in non-motile PKCβ-deficient T cells restored their locomotory behavior in response to an LFA-1 signal.

The possible involvement of PKCβ in IL-2 gene transcription and/or IL-2 protein secretion upon TCR/CD28-induced T cell activation has been addressed by several studies (Long et al., 2001; Dreikhausen et al., 2003). The downregulation of PKCβ synthesis in Jurkat T cells via the addition of antisense oligos resulted in the suppression of the activation of MAPK/NF-κB/NFAT pathways and a complete inhibition of IL-2 transcription and secretion. However, a study performed with a PKCβ-deficient HUT78 T cell clone excluded a possible role for IL-2 transcription and translation but demonstrated an involvement of PKCβ in IL-2 exocytosis. Thuille et al. (2004) investigated the physiological role of PKCβ in primary mouse T cells employing a PKCβ-deficient knockout line and found mostly normal activation-induced proliferation and IL-2 secretion responses. However, it is conceivable that other members of the cPKC family, such PKCα, could compensate for the lack of this redundant PKC isotype in T cells.

In 2010 a re-investigation of IL-2 expression in PKCβ silenced Jurkat T cells via antisense RNA technology revealed a stimulation dependent decreased IL-2 production, whereas the CD25 expression was significantly increased. In addition, PKCβ loss of function affected also CD69 surface levels and IL-8 production (Cervino et al., 2010). In the same year a scientific group investigated the influence of PKCβ on PMA induced apoptosis protection in Jurkat T cells and HL-60 human leukemia cells. The downregulation of PKCβ via shRNA or the specific small inhibitor enzastaurin reversed PMA induced protection of cell death (Meng et al., 2010).

### PKCα

Additional to PKCθ also PKCα, a member of the conventional PKCs plays an important role in the induction of a robust immune response. By transfecting fetal thymuses with constitutively active and dominant-negative forms of PKCα, Michie et al. (2001) showed that this isoform plays a specific role in the differentiation and expansion of immature thymocytes. Iwamoto et al. (1992) established a transgenic mouse line carrying rabbit PKCα cDNA under the control of the regulatory element of human CD2. In response to stimulation with anti-CD3, they found that the transgenic thymocytes proliferated extensively and produced IL-2 (Iwamoto et al., 1992). Lallena et al. (1999) and Trushin et al. (2003) showed that PKCα regulates IκB kinase and NF-κB in T cells.

PKCα was shown to be involved in the activation of the PI3K/Akt pathway, which is involved in T cell development, survival, and migration (Jones et al., 2000; Haxhinasto et al., 2008; Sauer et al., 2008). Using PKC inhibitors and *in vitro* kinase assays with recombinant inactive Akt as a substrate, Yang et al. (2006) showed that PKCα could phosphorylate Akt at Ser^473^ dependent on TCR activation. These authors also performed knockdown analysis in Jurkat T cells and found decreased TCR-induced phosphorylation of Akt at Ser^473^. PKCα and PKCθ are both involved in TCR downregulation (von Essen et al., 2006). von Essen et al. (2006) investigated the role of PKC isotypes in TCR downregulation and found an important role for PKCα in TCR comodulation (downregulation of non-engaged TCRs). Moreover, PKCα seemed to be responsible for the induction of endocytosis of non-engaged TCRs that recycle to the contact zone between the T cell and the APC. PKCθ, however, seemed to be responsible for inducing the endocytosis of directly triggered TCRs at the contact zone. Furthermore, a study showed the involvement of PKCα in allergic processes (Oh et al., 2004).

Our laboratory identified PKCα as a physiological and non-redundant PKC isotype in signaling pathways that are necessary for T cell-dependent IFNγ production and IgG2a/2b antibody responses using PKCα-knockout mice (Pfeifhofer et al., 2006).

## PKC LMWI (LOW-MOLECULAR-WEIGHT INHIBITOR) IN THE CLINIC

Studies have shown that PKCθ^-/-^ mice fail to develop experimental allergic encephalomyelitis (EAE) and display drastically reduced lung inflammation after the induction of allergic asthma and alloreactivity in TX medicine, suggesting that PKCθ by itself is an attractive monotarget for modulation of the immune response. While this published evidence validates PKCθ inhibition being essential, more recent results have indicated that additional PKC isotypes are involved in critical T cell signaling pathways. Because PKCθ and PKCα are both highly expressed in T cells (GNF SymAtlas (http://symatlas.gnf.org/SymAtlas) and have isotype-selective functions in T cells (Sun et al., 2000; Pfeifhofer et al., 2003, 2006), whether PKCθ and PKCα also exert overlapping functions has also been investigated. Gruber et al. (2009b) generated PKCα^-/-^θ^-/-^ double-knockout mice and found that the NFAT pathway plays a predominant role in the collaborative action of PKCθ and PKCα. The NFAT kinase GSK3β was hyper-reactive in PKCα^-/-^θ^-/-^ double-knockout CD3^+^ T cells. Subsequently, these authors found reduced nuclear translocation and DNA binding of NFAT. In *in vivo* studies, PKCα^-/-^θ^-/-^ double-knockout T cells showed strongly reduced IL-2 cytokine secretion after injection of an anti-CD3 monoclonal antibody. Additionally, the mice showed an impaired alloimmune response, leading to significantly prolonged allograft survival in heart transplantation experiments (Gruber et al., 2009b).

To obtain complete immunosuppressive effects, the inhibition of more than PKCθ appears to be needed, and the pharmacologic inhibition of multiple PKC isotypes may provide a successful approach to avert T cell effector functions that are relevant for diseases such as psoriasis, atopic dermatitis, and allergies, as well as other indications, including asthma, rheumatoid arthritis, multiple sclerosis, and transplant rejections.

Sotrastaurin (AEB071) is an immunosuppressive drug that inhibits multiple classical and novel members of the PKC family, resulting in decreased T lymphocyte activation (Evenou et al., 2009). In primary human and mouse T cells, AEB071 abrogated IL-2 secretion and CD25 expression, which are markers of early T cell activation. CD3/CD28-induced T cell proliferation, and LFA-1-mediated T cell adhesion were potently inhibited, and unlike previous PKC inhibitors, the apoptosis of murine T cell blasts was not enhanced (Evenou et al., 2009). These mechanistic studies on NF-κB and NFAT transcription factor transactivation additionally suggest that AEB071 and CsA have a complementary effect, resulting in the combined inhibition of IL-2 secretion. Additionally, other results suggest that AEB071 but not CsA inhibits the adhesive capacities of T lymphocytes.

Skvara et al. (2008) performed a clinical study with patients suffering from psoriasis in which the patients received single and multiple oral doses of AEB071. They found a strong reduction in the clinical severity of psoriasis and a histological improvement in skin lesions, indicating that sotrastaurin may provide a new therapeutic option for psoriasis (Skvara et al., 2008). Even so, we cannot exclude additional PKC isotypes being involved in critical T cell signaling pathways. The effect of AEB071 on PKCθ, including other classical and novel PKC family members expressed in T cells, is the likely mechanism responsible for the strong AEB071 immunosuppressive activity.

## NEW CANDIDATE EFFECTOR PATHWAYS MEDIATED BY PKC IN T CELLS

The challenge ahead for immunologists is the further elucidation of the molecular and cellular processes of PKCα and PKCθ that govern the development and function of T cells. PKC-mediated signaling in NFAT/AP-1 transactivation critically involves a pathway of the orphan nuclear receptor NR2F6. There is evidence that PKC-induced signaling involves NR2F6 inactivation, presumably by stimulating the release of NR2F6 from DNA-binding sites. This inactivation facilitates NFAT/AP-1 binding to its enhancers in the IL-2 and IL-17A promoters. In agreement, PKCα^-/-^/θ^-/-^ double-knockout T cells show almost no TCR/NFAT/AP-1 transactivation signaling (Gruber et al., 2009b), whereas NR2F6-knockout T cells show markedly upregulated TCR/NFAT/AP-1 transactivation (Hermann-Kleiter et al., 2008). However, PKCα and PKCθ might have an even broader role in regulating T cell functions than just acting downstream of T cell antigen receptors. Thus, despite the significant progress in assembling the PKC puzzle in T lymphocytes, defining downstream PKC substrates, including their effector functions, triggered by this phosphorylation step remains to be investigated in physiological settings. From these investigations, innovative possibilities are likely to emerge for the manipulation of T cell pathways in treating immunological diseases by suppressing pathophysiological immune responses or augmenting host-protective immunity.

## Conflict of Interest Statement

The authors declare that the research was conducted in the absence of any commercial or financial relationships that could be construed as a potential conflict of interest.
